# Generalized granuloma annulare as a paraneoplastic manifestation of acute myeloid leukemia and stage 1A malignant melanoma

**DOI:** 10.1016/j.jdcr.2025.07.011

**Published:** 2025-08-06

**Authors:** Evan Mak, Ford Lannan, Shena Kravitz, Stephen Bearman, Erik Kumetz, Aubrey Montebello, John Linabury, Liesl Grenier, Mariusz Wojnarski

**Affiliations:** a School of Medicine, Uniformed Services University of the Health Sciences, Bethesda, Maryland; b Dermatology Department, Walter Reed National Military Medical Center, Bethesda, Maryland

**Keywords:** acute myeloid leukemia, generalized granuloma annulare, melanoma

## Introduction

The relationship between dermatologic manifestations and systemic diseases is well recognized. Rare cases of granuloma annulare (GA) presenting as a manifestation of acute myeloid leukemia (AML) have been documented.^1^^,^^2^ We report a unique case of generalized GA presenting in a patient diagnosed with malignant melanoma and AML.

## Case report

**Fig 1:**
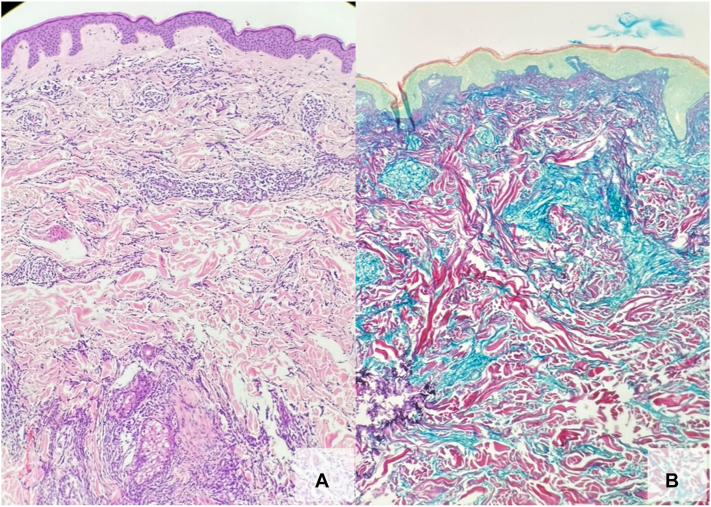
Punch biopsy of representative papule on the trunk showing features consistent with granuloma annulare. **A,** H&E: Histiocytes dissecting between dermal collagen bundles and grouping around dermal vessels and adnexal structures. Scattered CD3-positive T-cells and rare CD20-positive B-cells are present. CD117 and SOX10 immunohistochemical stains were negative. **B,** Alcian blue and colloidal iron special stains highlight increased interstitial mucin with aggregates of histiocytes palisading around the mucin. *H&E*, Hematoxylin and eosin.

**Fig 2:**
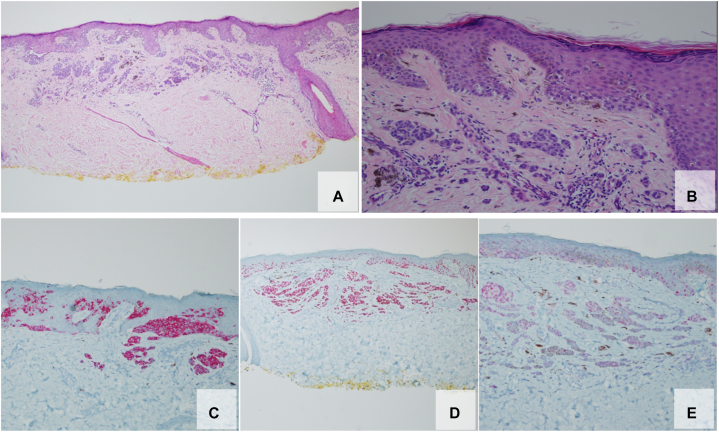
Shave biopsy of pigmented lesion consistent with melanoma. H&E **(A)** 40× and **(B)** 200× demonstrate an atypical melanocytic proliferation with severe architectural disorder and severe cytologic atypia. Confluent single-cell growth and focal pagetoid spread of melanocytes was confirmed on **(C)** Melan-A and **(D)** SOX10 stains. **E,** Junctional and dermal melanocytes were PRAME positive. *H&E*, Hematoxylin and eosin.

A 56-year-old Fitzpatrick skin type II male presented to the dermatology clinic with a 1-month history of round, pink, well-defined dermal papules and small erythematous plaques (0.8-1.5 cm) scattered on his chest and back. A skin punch biopsy of a representative lesion revealed CD68-positive spindled cells infiltrating the dermis in loose groups and aggregates, as well as increased interstitial mucin (Fig 1), supporting the diagnosis of GA. The patient was prescribed topical tacrolimus 0.1% ointment twice daily. At the same visit, an incidental finding of a 7-mm dark brown papule on the patient’s right shoulder prompted a shave biopsy, confirming stage 1A malignant melanoma (pT1a, Breslow depth of 0.7 mm) with histology shown in Fig 2.

**Fig 3:**
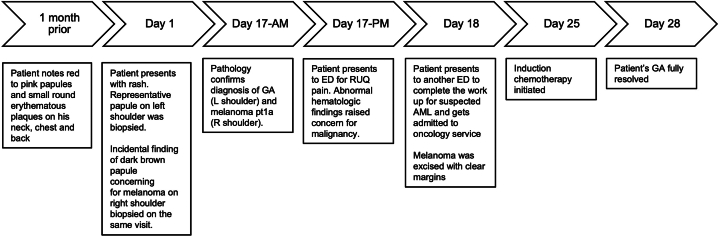
Timeline of events from the initial diagnosis of generalized GA to treatment and recovery. *GA*, Granuloma annulare.

Seventeen days after the initial visit for the GA rash, the patient presented to the emergency department with acute right upper quadrant abdominal pain. A complete blood count revealed 30% blasts on differential, leukocytosis (14,000/μL), and thrombocytopenia (90,000/μL). Additional findings included mild anemia with a hemoglobin of 12 g/dL (reference range: 13.5-17.5) and hematocrit of 36.8% (reference range: 41-53). Subsequent flow cytometry was remarkable for leukemic blasts expressing CD13, CD33, CD34, CD38, CD45 (diminished), CD117, CD71 (diminished), CD4 (minimal), and human leukocyte antigen-DR/intracellular myeloperoxidase. These findings led to a new diagnosis of AML. A bone marrow biopsy showed 55% blasts, decreased megakaryocytes, and erythroid precursors. Prior to induction chemotherapy for AML, the malignant melanoma was excised with 1.0-cm margins. The patient’s GA lesions resolved 10 days after melanoma excision and 3 days after initiating induction chemotherapy for AML with cytarabine (200 mg/m^2^, days 1-7), daunorubicin (900 mg/m^2^, days 1-3), and midostaurin (50 mg, days 8-21). GA has not recurred during the 15-month follow-up period after cessation of chemotherapy (Fig 3).

## Discussion

In this case, the appearance of GA lesions preceded the diagnoses of melanoma and the hematologic abnormalities indicative of AML by approximately 30 days. Previous reports have described granulomatous dermatitis associated with underlying malignancies, including lymphoma, leukemia, and solid organ tumors.^1^^,^^2^ However, there are limited data on the occurrence of GA in the context of malignant melanoma. GA in association with myelodysplastic syndrome and AML was first documented by Vestey et al.^2^ In their report, an elderly patient developed GA lesions that spread to the trunk and extremities, with AML confirmed via bone marrow biopsy.^2^

This case highlights a unique presentation where generalized GA appeared prior to symptoms, abnormal hematologic findings, or suspicion of AML. The melanoma was detected on examination 30 days after GA onset. Histologically, malignancy-associated granuloma annulare (MGA) shares some features with idiopathic GA, including histiocytic infiltrates, degenerating dermal collagen, and increased interstitial mucin.^3^ However, MGA is more likely to present with perivascular inflammatory infiltrates and occurs predominantly in the seventh decade of life. The idiopathic GA is more likely to exhibit increased mucin, multinucleated giant cells, and affects younger populations. In this case, the histologic findings were consistent with idiopathic GA, but the temporal relationship and resolution following AML treatment and melanoma excision align with Curth’s postulate for paraneoplastic phenomena.

The role of noninvasive melanoma in the development of GA remains unclear. It is hypothesized that tumor antigens may stimulate an immunologic reaction leading to GA.^4^ In this case, the immunological response to both AML and melanoma possibly contributed to GA lesions. It has been postulated that tumor cells may cause decreased cell-mediated immunity and secretion of cytokines that contribute to an inflammatory response that mediates granuloma formation.^4^ Reports of dramatic clinical response of GA to treatment with tumor necrosis factor-α inhibitors also highlight the possible role of malignancy-induced immunodeficiency.^4^^,^^5^ In addition to malignancy, individuals with deficiencies in cell-mediated immunity are observed to develop GA in greater frequency.^4^ This supports the hypothesis that immunologic factors may play a role in MGA.^4^ The hematologic abnormalities seen in AML and immunological response seen in melanoma might have synergistically contributed to a malignancy-induced immunodeficient state with tumor-mediated cytokines and inflammatory activation that mediated granuloma formation.

Similar to the immunosuppressive effects of standard GA treatments, the immunosuppressive effects of the patient’s chemotherapy may have contributed to the GA resolution. A case-control study found all paraneoplastic GA cases improved following definitive treatment of the associated solid organ malignancy, including resolution 1 month after gastrointestinal stromal tumor removal.^5^^,^^6^ However, our patient’s rapid GA resolution within 3 days of chemotherapy initiation differs from typical AML immune responses, which occur after 3 to 4 weeks^7^^,^^8^ This suggests that melanoma excision may have contributed to GA resolution, although AML-directed treatment via cytoreductive and anti-inflammatory effects was likely the primary driver, warranting further investigation.

This report supports the growing evidence that generalized GA may serve as a paraneoplastic dermatologic manifestation of malignancies beyond lymphoproliferative disorders and solid organ tumors.^9^ Notably, previous reports of GA associated with melanoma have involved metastatic disease and systemic therapies such as BRAF inhibitors or immune checkpoint inhibitors.^10^ Our case is the first report of generalized GA in stage 1A melanoma without confounding systemic treatment for metastatic melanoma.

Clinicians should recognize the potential association between generalized GA and malignancy. In cases of unexplained or refractory GA, thorough history and evaluations for lymphoproliferative disorders, hematologic malignancies, solid organ tumors, and skin examination for melanoma should be considered in addition to standard screenings (eg, HbA1c, lipid profiles, thyroid function tests).^9^ The rapid resolution of GA within 3 days of initiating treatment for AML and 10 days post excision of melanoma underscores the possible correlation. This is consistent with other reports of rapid GA resolution within 1 month of initiation of treatment for malignancy.^6^ The individual contribution of AML and melanoma to the development of generalized GA lesions remains to be determined in future studies. Further research is warranted to investigate a possible mechanistic connection between GA, AML, and melanoma.

## Conflicts of interest

None disclosed.
